# Phytochemical-Based Nanomaterials against Antibiotic-Resistant Bacteria: An Updated Review

**DOI:** 10.3390/polym15061392

**Published:** 2023-03-10

**Authors:** Rocío Díaz-Puertas, Francisco Javier Álvarez-Martínez, Alberto Falco, Enrique Barrajón-Catalán, Ricardo Mallavia

**Affiliations:** 1 Instituto de Investigación, Desarrollo e Innovación en Biotecnología Sanitaria de Elche (IDiBE), Universidad Miguel Hernández (UMH), 03202 Elche, Spain; 2 Instituto de Investigación Sanitaria y Biomédica de Alicante (ISABIAL), 03010 Alicante, Spain

**Keywords:** nanotechnology, nanofibers, nanoparticles, green synthesis, electrospinning, plants, antimicrobial

## Abstract

Antibiotic-resistant bacteria (ARB) is a growing global health threat, leading to the search for alternative strategies to combat bacterial infections. Phytochemicals, which are naturally occurring compounds found in plants, have shown potential as antimicrobial agents; however, therapy with these agents has certain limitations. The use of nanotechnology combined with antibacterial phytochemicals could help achieve greater antibacterial capacity against ARB by providing improved mechanical, physicochemical, biopharmaceutical, bioavailability, morphological or release properties. This review aims to provide an updated overview of the current state of research on the use of phytochemical-based nanomaterials for the treatment against ARB, with a special focus on polymeric nanofibers and nanoparticles. The review discusses the various types of phytochemicals that have been incorporated into different nanomaterials, the methods used to synthesize these materials, and the results of studies evaluating their antimicrobial activity. The challenges and limitations of using phytochemical-based nanomaterials, as well as future directions for research in this field, are also considered here. Overall, this review highlights the potential of phytochemical-based nanomaterials as a promising strategy for the treatment against ARB, but also stresses the need for further studies to fully understand their mechanisms of action and optimize their use in clinical settings.

## 1. Introduction

Bacterial infections pose a major threat to human health worldwide, especially when resistant to conventional antibiotics. In 2019, over 4.95 million fatalities worldwide were associated with antimicrobial resistance (AMR) illnesses, among which 1.27 million were directly linked by them, i.e., fatalities that could have been prevented if the infections had been susceptible to antibiotics, thereby becoming a leading cause of death worldwide in low-resource environments [1]. Due to the alarming appearance of antibiotic-resistant bacteria (ARB) on multiple antibiotics, their rapid spread, and the slow discovery of new antibiotics, conventional therapies are gradually losing effectiveness [2]. There are several factors contributing to the development of ARB, including: (i) the overuse and the misuse of antibiotics [3], often as a consequence of a lack of new ones [4], (ii) poor infection control measures [5], (iii) genetic factors, and (iv) environmental factors [6].

Alternative and complementary treatments to antibiotics have been steadily pursued in the last few decades to address this issue [7]. In this sense, it is predicted that natural sources still harbor a huge number of bioactive molecules that are yet to be discovered, particularly within plants (kingdom *Plantae*) [8]. Plant extracts can contain a wide variety of phytochemicals such as polyphenols, alkaloids, and terpenoids with proven antibacterial capacity, even against ARB [9,10]. Phytochemicals are usually less potent than traditional antibiotics, although often endowed with therapeutically interesting properties such as molecular promiscuity or AMR-modifying capacities. Not in vain, plant extracts have been used by human communities since ancient times, when scientific knowledge was practically nil and only reduced to trial-and-error screenings [11]. Nowadays, the development of modern technology can help to optimize the use of these phytochemicals and to enhance their benefits for human health, as is the case for nanotechnology.

In recent years, the use of nanotechnology in biomedical applications has been increasing rapidly, observing a pronounced upward trend in the number of scientific articles published in this regard. Nanomaterials such as nanofibers (NFs) and nanoparticles (NPs) can tackle limitations related to traditional approaches [12] and provide beneficial morphologies and surface features to fight bacteria [13]. In addition, the characteristics of these nanomaterials, i.e., size, shape, constituents, and surface, can adjust their mechanical, biological, and physicochemical properties to match the required needs [14]. The polymeric matrices of nanomaterials can result in desirable characteristics, such as small size and high surface-to-volume ratio. These properties can enhance the permeability and solubility of drugs encapsulated within them, making them ideal for drug delivery. Phytochemicals could potentially utilize these features to exert their antimicrobial effects [15]. These features can also improve the biopharmaceutical properties of the final products, with a special interest on low bioavailable compounds [16].

The present study reviews the recent advancements in the development of polymeric NFs and NPs loaded or synthesized with phytochemicals as promising tools to fight against ARB.

## 2. Study Design

The Scopus and MedlinePlus databases were used to perform a bibliographic search using the following keywords: “nanomaterials” OR “nanoparticles” OR “nanofibers” AND “antibacterial” AND “resistance” AND “plant extract” OR “essential oil” OR “phytochemical”. Among the 378 results retrieved, about 77% (224) corresponded to publications from the last 5 years. A selection was made among the publications from 2014 onwards. The inclusion criteria for articles were: (i) published in English; (ii) in peer-reviewed journals; and (iii) focused on the use of phytochemical-based nanomaterials for the treatment of ARB. A total of 170 articles were included in the final review. From those, 34 publications were experimental studies focused on phytochemical-based nanomaterials against ARB, from which 18 belonged to the last two years. The remaining 136 publications were included in the body of the article, i.e., Introduction and other sections not directly related to experimental studies.

## 3. Antimicrobial Capacity of Phytochemicals

As briefly mentioned above, poultices and infusions have been prepared from local plants for medicinal purposes since ancient times, including curing bacterial infections [11]. Since the discovery and implementation of antibiotics in the middle of the 20th century, the use of plants as antimicrobials has been drastically reduced. However, the rise of ARB has pushed researchers to search for new antimicrobial compounds from various sources, thus revisiting the plant world as it represents a large reservoir of bioactive molecules with therapeutic potential yet to be explored in depth [17].

**Figure 1:**
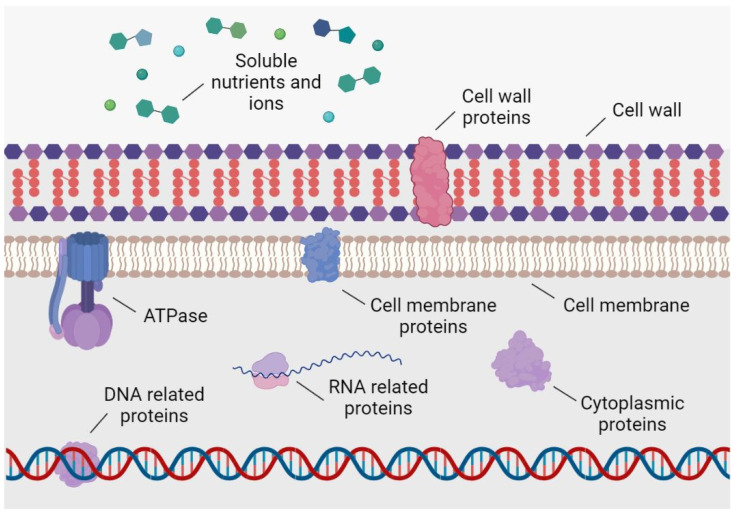
Main bacterial molecular targets of phytochemicals with antibacterial activity.

One of the main advantages of the use of phytochemicals for antimicrobial purposes is their multifactorial capacity or molecular promiscuity. While traditional antibiotics usually act on a specific bacterial molecular target with great efficacy, phytochemicals can bind to several, but generally with less affinity than that of antibiotics. Such promiscuity or multitarget affinity potentially hinders the generation of possible resistance mechanisms in the bacteria [18]. As it is schematized in Figure 1, the different bacterial molecules targeted by phytochemicals include cell wall [19] and the cell membrane components [20] as well as proteins with diverse locations and functions [21], and thus with the ability of even interfering in the nutrient metabolism and motility [22]. In addition to these pharmacological direct activities, it has been shown that there are phytochemicals, such as certain polyphenols, that are capable of sensitizing ARB by reversing their resistance mechanisms and making them more susceptible to traditional drugs [23,24]. 

Phytochemicals may also act in synergy with antibiotics, such as antibiotic adjuvants [25], enhancing their antimicrobial activity and potentially reducing the amount of antibiotic needed to treat an infection [26]. This combination may help to slow the development of AMR, as well as to minimize their adverse effects and environmental impact [27]. Additionally, phytochemicals may help to boost the immune system and improve the overall health of an individual, conferring protection to infection [28].

To date, the literature on AMR to phytochemicals is limited. One example is a study linking genetic changes in *Lysteria monocytogenes* (deletion of the *sigB* gene) with increased resistance to carvacrol, thereby showing that it is feasible for bacteria to develop resistance to specific polyphenols with unique or few molecular targets [29]. Another example of AMR to botanicals is the presence of “tannin-resistant” Gram-positive bacteria (*Streptococcus* sp.) at sites of high exposure to these polyphenols, such as goat, sheep, and deer rumens [30]. This type of bacteria is thought to protect ruminants from possible tannin anti-nutritional dietary influences [31]. Mechanisms by which bacteria overcome the inhibitory effects of tannins on growth include substrate modification, dissociation of tannin-substrate complexes, formation of extracellular polysaccharides, cell membrane modification, and metal ion chelation [32]. Importantly, bacteria prevalent in ruminant gastrointestinal tannin media may not themselves be resistant. This resistance may be more related to improve the nutrient accessibility of bacteria in the special microenvironment of the ruminant stomach [33]. However, drug resistance seems unlikely to develop when complex mixtures of polyphenols affecting multiple molecular targets on bacterial cells are used [34,35], and so it occurs for plant extracts that contain an amalgam of phytochemicals [36].

One of the main limitations for the use of phytochemicals as antibacterial agents is the low availability and poor pharmacokinetic properties. Widely studied phytochemicals such as quercetin [37] and curcumin [38] present these limitations. The use of drug delivery systems, such as nanomaterials, could help to overcome these limitations [39]. In the following sections, the combinations of phytochemicals and different types of nanomaterials will be described.

## 4. Nanofibers

NFs are one-dimensional nanomaterials whose properties make them suitable for a wide range of applications, including drug delivery [40,41], tissue engineering [42], water/air filtration [43], energy storage [44], protective clothing [45], sensors or photocatalytics [46], among others [47,48]. One of the key features of NFs is their large surface area to volume ratio, which allows them to interact with their surroundings in ways that are not possible with larger fibers [49]. This can make them more effective at adsorbing or filtering small molecules or particles [50].

NFs can be prepared from natural or synthetic polymers, metals, ceramics, semiconducting, composite, and carbon-based materials [51]. Synthetic and natural polymers are particularly used in the synthesis of NFs for biomedical applications due to their biocompatibility, biodegradability, and processability [13,52]. Synthetic polymers include polyethilene glycol (PEG), a water-soluble biocompatible polymer with good drug-carrying capacity [53]; polyvinyl alcohol (PVA), a water-soluble biocompatible polymer [54]; polyvinylpyrrolidone (PVP), a water-soluble polymer with high biocompatibility [55]; polycaprolactone (PCL), a biocompatible and biodegradable polymer [56]; polylactic acid (PLA), a biocompatible, biodegradable polymer that is often used in drug delivery applications [57]; or polyethyleneimine (PEI), a cationic polymer with good drug-carrying capacity and ability to evade the immune system [58]. The most widely used natural polymers for the synthesis of NFs in drug delivery applications are chitosan (CS), a biocompatible, biodegradable polymer derived from chitin [59]; gelatin, a protein derived from collagen, which is a natural polymer found in connective tissue [60]; alginate, a natural polymer derived from brown seaweed, brown algae (Ochrophyta, Phaeophyceae) and bacteria (*Azotobacter vinelandii* and *Pseudomonas* species) [61]; hyaluronic acid, a natural polymer found in connective tissue [62]; or dextran, a natural polymer derived from glucose [63]. Natural polymers are biocompatible and have good drug-carrying capacity, making them useful in drug delivery and tissue engineering applications [64]. Overall, the choice of polymer for NF synthesis for drug delivery applications depends on the specific requirements of the application, including the desired drug-carrying capacity, biocompatibility, and other factors. As for other nanomaterials, it is also important to consider the intended route of administration and the stability of the drug in the polymer matrix [65].

### 4.1. Synthesis of Polymeric NFs

Polymeric NFs can be synthesized using different technologies, such as electrospinning, self-assembly, template-based synthesis, polymerization or sonochemical synthesis [51]. Among the different methods that exist to produce them, electrospinning is the most used because it is simple, cheap, versatile, reproducible, and scalable [66]. Recently, the term “green electrospinning” has emerged as a method for synthesizing NFs using environmentally-friendly and sustainable materials and processes. It involves the use of biodegradable, biocompatible, and renewable materials, as well as energy-efficient and low-waste production methods. It is based on the use of natural or biosynthetic biodegradable polymers and the use of non-toxic solvents [67].

Electrospinning allows NFs to be created by loading and expelling a polymer solution through a needle subjected to a high-voltage electric field. The solution with the desired polymer is drawn into a syringe attached to a needle and pumped at low speeds until a drop forms at the tip of the needle. Subsequently, the solution is subjected to a high electrical charge produced by a high voltage source. As the voltage increases, the drop at the tip of the needle begins to deform until it begins to exert a magnitude of force, such as the surface tension of the solution itself. At this time, a cone shape with convex sides and a rounded tip, known as Taylor cone, begins to form [68]. When a certain voltage threshold is reached, a jet of liquid begins to be emitted. During the movement of the jet between the needle and the collector, the solvent evaporates and a solid polymer fiber is collected. The collector is also connected to the high voltage source and is usually made of conductive metal [69]. 

**Figure 2:**
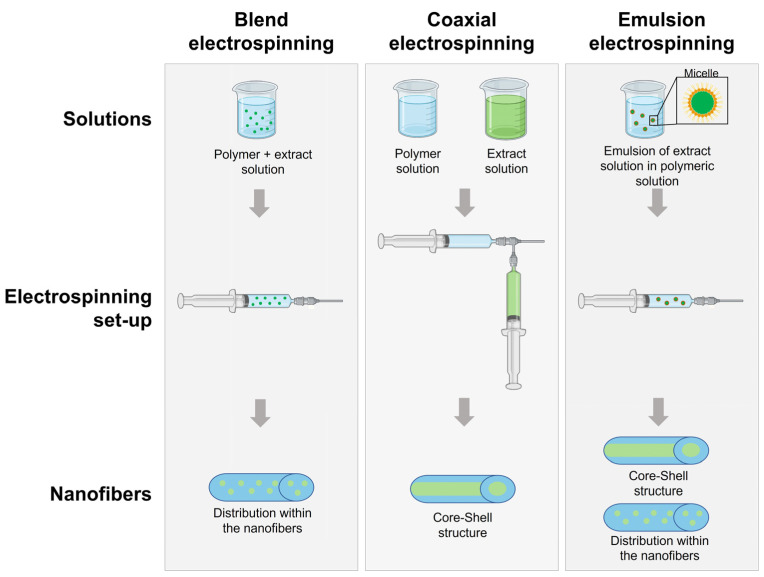
Diagram of electrospinning process for the manufacturing of NFs loaded with plant extracts.

Drug encapsulation in NFs can be performed by electrospinning using methods such as blend electrospinning, coaxial electrospinning, and emulsion electrospinning (Figure 2). In blend electrospinning, the drug is mixed with the polymeric solution before the electrospinning process. Therefore, the drug is expected to be dispersed in the polymeric matrix and uniformly distributed in the NFs [70]. Coaxial electrospinning is based on the co-spinning of two solutions using two needles located coaxially, one with the polymeric solution and the other with the therapeutic solution. Core-shell fibers are obtained, where normally the polymeric matrix is found in the outer core and the therapeutic agent is incorporated in the inner core [13]. Emulsion electrospinning solutions are based on two or more immiscible liquid phases that will be electrospun together using the same set up as blend electrospinning [70]. The distribution of the compounds in the NFs depends on their molecular weight. It has been observed that high molecular weight compounds tend to form core-shell structures, while low molecular weight compounds are distributed throughout the NFs [71].

### 4.2. Antibacterial Properties of Polymeric NFs

NFs can exert their antibacterial activity per se through a variety of mechanisms depending on the specific properties of the NFs, polymers used, and the type of bacteria being targeted. NFs with a small pore size or high surface area can physically entrap bacteria, preventing them from growing or spreading [72]. The surface chemistry of NFs can also affect their ability to interact with bacteria. For example, NFs with a positive charge may be able to attract and kill negatively charged bacteria, while those with a hydrophobic surface may be able to inhibit the growth of hydrophilic bacteria [13]. NFs can be designed to release antimicrobial agents, which can kill or inhibit the growth of bacteria [73]. NFs can also stimulate the immune system, helping to fight off bacterial infections [74]. In addition, by utilizing a polymer with antimicrobial capabilities, such as CS, NFs can exhibit their antibacterial activity. The antibacterial activity of CS can be attributed to its adsorptive characteristics to bacterial cells due to electrostatic interactions between the polycationic structure of CS and the anionic groups found on the bacterial cell surface [75]. This causes permeabilization of the cell membrane and the loss of essential constituents as enzymes, nucleotides, ions, and death of the bacterial cell.

### 4.3. Plant-Based NFs against ARB

The use of phytochemicals for the formulation of polymeric NFs is in continuous development and has attracted attention especially for their enhanced antimicrobial and wound healing activities [76]. Therefore, these formulations can be promising alternatives to treat ARB infections. The combinations of polymers and phytochemicals for the synthesis of loaded NFs with antimicrobial application against ARB are summarized in Table 1.

Thamer et al. (2022) recently fabricated PVA/tragacanth gum (TG) NF mats loaded with an aqueous extract of myrrh (myrrh@PVA/TG NFs) [81]. Results showed that 15%-myrrh@PVA/TG NFs had a mean diameter of 220 nm and displayed antibacterial activity against drug-resistant (DR) *S. aureus* with 13.33 mm of inhibition zone. In a study by Ramalingam et al. (2021), core-shell NFs were synthesized by coaxial electrospinning using PCL/gelatin as shell structure [78]. *G. sylvestre* leaf extracts were added to the core and the antibiotic minocycline hydrochloride was added to the shell. Results prior to the synthesis of NFs showed synergism between plant extracts and minocycline against Gram-positive bacteria. The incorporation of *G. sylvestre* extract to the PCL/gelatin solution resulted in a reduction in the diameter of the ensuing NFs from 443 to 302–340 nm. In addition, the NFs were able to inhibit MRSA in a disc diffusion assay with inhibition zones of 15.2–19.1 mm. 

There are many different polymers used to manufacture NFs, with PVP and PCL being the only ones used in combination with more than one phytochemical among the selected studies. These polymers are widely used in biomedicine thanks to their particular properties. PVP is water-soluble, pH-stable, temperature-resistant, non-toxic, biodegradable, and biocompatible [87]. PCL, a polyester, has been employed extensively in the field of tissue engineering due to its accessibility, reasonable cost, and appropriateness for modification. It can be utilized under difficult mechanical, physical, and chemical conditions without suffering a major loss of its qualities because its chemical and biological properties, physicochemical state, degradability, and mechanical strength can all be altered [56]. 

In the selected studies, the most used technique for the fabrication of NFs loaded with phytochemicals against ARB was blend electrospinning, most likely because it is a simpler process compared to coaxial or emulsion electrospinning, which can be more complex and require specialized equipment [88].

There is a great variety in the phytochemicals used in the NFs, curcumin being the only one that appears in different studies. Numerous investigations revealed that curcumin had antibacterial effects on both Gram-positive and Gram-negative bacteria. Curcumin’s antibacterial properties include bacterial membrane rupture, oxidative stress induction, suppression of bacterial virulence factor synthesis, and biofilm formation [89]. These qualities also help to explain why curcumin functions as a broad-spectrum antibacterial adjuvant, as demonstrated by the substance’s pronounced additive or synergistic interactions with numerous conventional antibiotics and non-antibiotic substances [90].

In some studies, the addition of phytochemicals or their concentration increase resulted in a reduction in the diameter of the fibers [79,81,82,84]. This phenomenon is attributed to the higher charge density and conductivity. Previous studies indicate that a higher charge density in the Taylor cone makes its radius of curvature smaller, causing a concentration of electrical stress at the tip. This occasions the initial jet to emerge from a smaller area and mass deposition decreases [91]. Therefore, the addition of phytochemicals can be advantageous, since smaller diameters more similar to the size of the bacteria can enhance bacterial attachment and inhibition [92].

Several types of NFs containing phytochemicals have been shown to have antibacterial activity against ARB. MRSA is the most studied bacteria in antibacterial assays employing NFs containing phytochemicals. Its widespread prevalence and ability to cause a range of infections, as well as its AMR to antibiotics, make MRSA a useful model organism for studying antibacterial agents and mechanisms of action [93]. 

Most assays employed to determine antibacterial activity of NFs were Kirby–Bauer. This can lead to difficulties when comparing different studies, since although the diameter of the inhibition zone is provided, in many cases data on the mass of NFs used for the test are not disclosed. Thus, there is a lack of information on the amount of phytochemicals released into the medium. This fact makes it difficult to compare the results with other studies that provide specific minimum inhibitory concentrations (MIC) [94].

## 5. Nanoparticles

NPs represent a relatively new strategy to tackle ARB. Its unique characteristics are a result of its physical properties, which are often interrelated. Their very high specific surface area results in enhanced reactivity, increased solubility in certain solvents, improved drug-carrying capacity and enhanced catalytic activity [95]. In addition, they have high mobility in free state and in porous media, which can allow them to more easily reach their target site, interact with their surroundings, disperse in a particular medium and increase their sensitivity to certain factors, such as temperature or pH [96]. Finally, NPs of 10 nm or less exhibit quantum confinement effect due to the restriction of charge carrier motion to a small volume [95]. The quantum confinement of electrons in NPs can enhance optical and electronic properties and increase stability and reactivity [97].

NPs can be classified into carbon-based, metal, ceramics, semiconductor, polymeric and lipid-based NPs [98]. Among them, polymeric and metal NPs (MNPs) are widely used for antimicrobial applications. Polymeric NPs offer advantageous properties for the encapsulation of antimicrobial drugs, such as controlled release of antimicrobial agents [99], target delivery [100], biocompatibility [101], improvement of bioavailability [102], possibility of reducing the administered dose [103], permanence in the circulatory system for longer periods [104] and customizability [105]. On the other hand, MNPs have shown to possess bactericidal capacity by themselves using mechanisms that will be described in subsequent sections. During the last few years, a multitude of phytochemicals have been used for the green synthesis of MNPs [106].

### 5.1. Polymeric NPs

Polymeric NPs can be made of synthetic or natural polymers. Synthetic polymers employed for NPs synthesis include PVA, poly(N-isopropylacrylamide) (PNIPAM), poly(lactic-co-glycolic acid) (PLGA), PLA, PCL, or PEG [65]. Natural polymers include CS, alginate, albumin, hydroxyapatite, pectin, or hyaluronic acid [107]. Polymeric NPs can also be classified into nanospheres and nanocapsules, depending on their morphological structure. Nanospheres are formed by a solid polymeric framework with a spherical structure in which the drugs are embedded or attached to its surface. Nanocapsules are formed by a polymeric shell that surrounds an interior space where the drugs of interest are located [108]. In addition, nanocapsules are typically smaller and have a higher drug-carrying capacity and a slower release rate compared to nanospheres [109].

#### 5.1.1. Synthesis of Polymeric NPs

**Figure 3:**
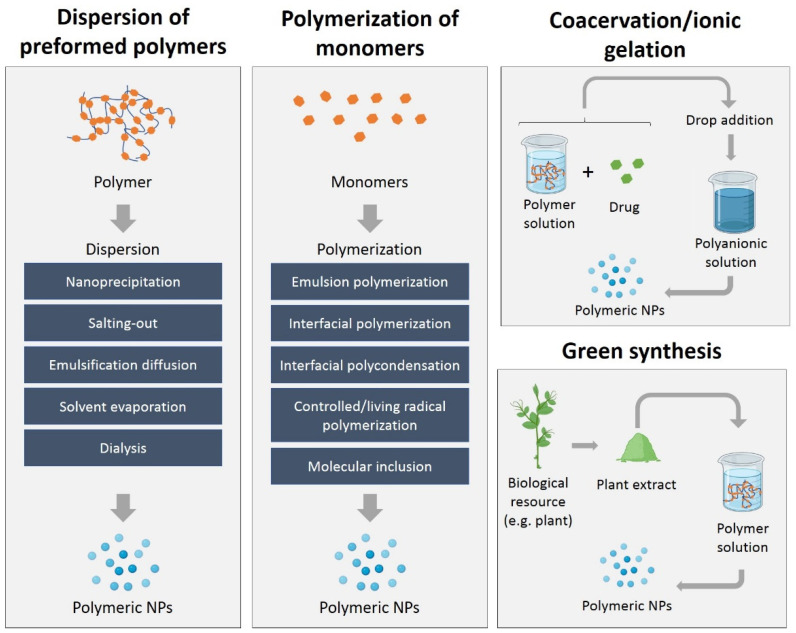
Schematic diagram of the different methods of synthesis (dispersion of preformed polymers, polymerization of monomers, coacervation/ionic gelation, green synthesis) of polymeric NPs.

Three main methods can be used to create polymeric NPs: the dispersion of preformed polymers, polymerization of monomers, and ionic gelation/coacervation of hydrophilic polymers. The different methods for the synthesis of polymeric NPs are described in Figure 3. The synthesis strategy will depend on the type of drug to be encapsulated, its administration, area of application, or size requirements, among other factors [15]. 

In the synthesis by polymerization of monomers, the NPs are synthesized by techniques such as emulsion polymerization, interfacial polymerization, interfacial polycondensation, controlled/living radical polymerization, or molecular inclusion [15]. Polymeric NPs can also be synthesized by the dispersion of preformed polymers using techniques such as nanoprecipitation, salting-out, emulsification diffusion, emulsification evaporation, and double emulsion solvent evaporation [110]. 

The ionic gelation/coacervation method is based on the mixture of a polymer and a coacervating agent, which is usually a polyelectrolyte. As the coacervating agent is added, it begins to interact with the polymer, causing the polymer chains to coacervate and form NPs. The size and shape of the NPs can be controlled by adjusting the concentration of the polymer and coacervating agent, as well as the pH and temperature of the solution [111]. 

Recently, the synthesis of chitosan nanoparticles (CSNPs) using biological systems was described for the first time. This new methodology is based on the mixture of a chitosan solution at 1.08% with an extract solution of *Pelargonium graveolens* and its incubation for approximately 1 h at 50 °C. The plant *P. graveolens* was selected as a bioconverting agent due to its nonhazardous and environmentally friendly character [112].

#### 5.1.2. Antibacterial Properties of Polymeric NPs

Polymeric NPs have suitable characteristics for use in drug delivery applications of antimicrobial agents, such as low toxicity, biocompatibility, biodegradability, or surface modification for specific targeting, among others [108]. These characteristics make it possible to improve the therapeutic index of drug loads in this type of system and to carry out a therapy focused on the infection site [113]. Some polymers, such as CS, have intrinsic antimicrobial activity, thus being able to kill bacteria or inhibit their growth by disrupting their cell membranes or inhibiting their metabolism [75]. Polymeric NPs can also coat surfaces and prevent bacteria from adhering to them. This can be useful for preventing the formation of biofilms, which are layers of bacteria that can be difficult to remove [114]. When exposed to light, polymeric NPs can produce reactive oxygen species (ROS), which can damage bacterial cells and inhibit their growth, thus being beneficial for photodynamic therapy, a type of treatment that uses light and photosensitizers to kill bacteria [115]. Polymers can also be modified to incorporate cationic and hydrophobic moieties such as peptides, small molecules, carbohydrates, antibodies, proteins, nucleic acids or antimicrobial drugs. This facilitates entry into the bacterial membrane, since cell walls are normally negatively charged [116]. Another possible mode of action of polymeric NPs is through the release of antimicrobial agents. Polymeric NPs can be loaded with antimicrobial agents, which are then released in a controlled manner when the NP comes into contact with the bacterial cell [117]. This can enhance the efficacy of the antimicrobial agent by allowing it to accumulate at the site of infection for a longer period of time [118].

There are several parameters that affect the antimicrobial activity of polymeric NPs, for example, crosslinking, micellization, molecular weight, polymer type and concentration, size, surface area, surface chemistry, or surface charges. The effects of these parameters on the antimicrobial activity can be seen in Table 2.

#### 5.1.3. Plant-Based Polymeric NPs against ARB

The use of polymeric NPs loaded with phytochemicals offers a promising strategy for the treatment against ARB, as it combines the antimicrobial activity of the phytochemicals with the controlled release and targeted delivery capabilities of the nanoparticles. Table 3 summarizes studies focused on polymeric NPs loaded with phytochemicals against ARB. 

Jamil et al. (2016) encapsulated cardamom essential oil (EO) in CSNPs using ionic gelation process. The size of the NPs was 50–100 nm and their antibacterial activity was tested against MRSA and ESBL *E. coli*. CSNPs loaded with cardamom EO could control bacterial growth for up to 7 days, while empty CSNPs could only maintain their antibacterial activity for 48 h [127].

Recently, CSNPs loaded with *E. globulus* leaf extract were synthesized via green synthesis method. The polymeric NPs were spherical with a diameter ranging 6.92–10.10 nm. Their antibacterial activity was tested against biofilm forming *A. baumanii,* and zones of inhibition of 12, 16, and 30 mm were recorded using concentrations of 12.5, 25, and 50 mg/mL, respectively. In addition, damage to the bacterial cell membrane, leaks of the cytoplasmic content into the extracellular medium, and the appearance of coagulations in the cytoplasm were observed [128]. 

CS is the most used polymer to make polymeric NPs loaded/synthesized with phytochemicals against ARB among studies included in Table 3. CS is a popular polymer for making NPs because of its biocompatibility and biodegradability [131]. This means that it is non-toxic to living cells and can be safely disintegrated by the body after it is no longer needed. CS can also form stable NPs with a range of different compounds, including both hydrophilic and hydrophobic molecules [132]. In addition, CS is a relatively inexpensive and widely available material, which makes it an attractive choice for use in NPs production [133]. Overall, the combination of these properties makes CS a popular choice for making polymeric NPs in a variety of applications. 

The heterogeneity in the rest of the parameters included in Table 3, as well as the scarcity of existing studies that use phytochemical-loaded polymeric NPs against ARBs, hinder the direct comparison of the remaining information. 

### 5.2. Metal NPs

MNPs are nanomaterials formed from pure metals (eg Au, Ag, Cu, Fe, Pt, Pd, Ti, or Zn) or their compounds (e.g., CuO, Fe_3_O_4_, TiO_2_, ZnO) and have dimensions in the nanometer range (1–100 nanometers). MNPs have unique physical and chemical properties that are different from those of bulk metals, due to the influence of their small size and high surface area-to-volume ratio [95]. Currently, they are widely used in biomedical sciences and engineering due to their enhanced properties, such as high mechanical and thermal stability, high surface area, and high optical and magnetic properties [134].

#### 5.2.1. Synthesis of MNPs

MNPs synthesis methods can be divided into two types: top-down and bottom-up approaches. Top-down approaches use destructive methods to break down a larger molecule into nanometer-sized particles in successive steps. This can be achieved through techniques such as grinding, attrition, sputtering, and laser ablation, among others [135]. Bottom-up approaches refer to methods in which MNPs are formed from simpler substances by self-assembly. Methods based on chemical reactions are widely used in this approach. Some examples are sol-gel, physical/chemical vapor deposition, spray/flame pyrolisis, chemical redction, hydrothermal/solvothermal methods, or biological methods [95].

**Figure 4:**
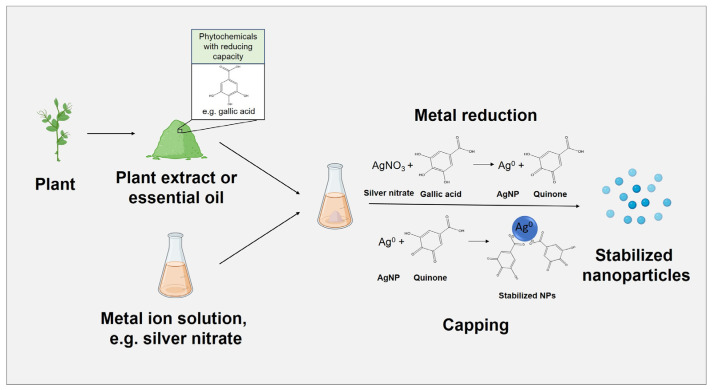
Schematic representation of the methodology used in the green synthesis of metallic nanoparticles using plant extracts.

MNPs synthesis can also be classified according to the generation method. Thus, they can be generated by physical, chemical, and biological approaches. The physical methods of synthesis of MNPs mostly employ top-down strategies. Although physical and chemical can produce high purity MNPs, elevated energy consumption or the use of toxic chemical agents limit the applications of these methods [136]. Biological or “green” synthesis employs biological routes from bacteria, fungi, or plants for the synthesis of NPs [106]. Some of its advantages include the minimization of waste, the use of safer solvents as well as renewable feedstock, its simplicity, and its cost-effectiveness [137]. Plants are especially used for the synthesis of NPs, since they are non-pathogenic and have biomolecules, such as proteins, amino acids, polysaccharides, terpenes, alkaloids, phenolics, saponins, and vitamins [106], capable of reducing and stabilizing metal salts into NPs (Figure 4). The presence of MNPs within living plants after uptake of metal ions has been reported in species such as *Aloe vera*, *Medicago sativa*, or *P. graveolens*, among many others [138].

#### 5.2.2. Antibacterial Properties of MNPs

Although the exact mechanism of action of MNPs in bacterial cells remains unknown, some mechanisms have been proposed over the years. These include: the release of ions, which can disrupt the bacterial cell membrane and inhibit bacterial growth [139]; the production of ROS within microorganisms, damaging bacterial cells and inhibiting their growth [140]; the disruption of vital enzymes of the respiratory chain through microbial plasma membranes, damaging [141] or physically damaging bacterial cells due to their size and shape [142]. The mechanism of action of AuNPs against *E. coli* was studied by Cui et al. (2012). They demonstrated that AgNPs were capable of membrane collapse potential, inhibiting ATPase activities to decrease the ATP level, and inhibiting the subunit of ribosome from binding tRNA [143]. The ability of AgNPs to inhibit *S. aureus* bacterial growth was investigated by Li et al. (2011). They found that AgNPs were able to cross the cell wall and interfere with cell metabolism from the cell membrane. They were also able to cross the membrane and condense DNA to prevent it from replicating and cells from reproducing, producing the subsequent bacterial destruction [144].

#### 5.2.3. Plant-Based MNPs against ARB

The use of plant parts, such as extracts or EOs from leaves, fruits, roots, stems, or seeds for the biosynthesis of NPs in vitro is being widely studied due to the biocompatibility, safety, and environmental harmlessness that this method presents [145]. The use of MNPs in combination with phytochemicals can be an effective strategy to combat ARB. Although there is evidence that bacteria can develop resistance strategies against MNPs, their nonspecific mode of action toward multiple cellular components suggests that development of resistance is less likely to occur compared to traditional antibiotics [146]. The use of phytochemicals to synthesize MNPs involves a more environmentally friendly approach for the fight against ARB. Table 4 shows NPs synthesized using phytochemicals for antimicrobial applications against ARB.

Among the MNPs used in in vitro antibacterial activity assays against ARB, AgNPs are the most studied. Tyavambiza et al. (2021) used *C. orbiculate* leaf extract, a succulent plant indigenous to Southern Africa, to synthesize AgNPs. The AgNPs were formed using three different concentrations, 6, 3, and 1.5 mg/mL, whose mean diameters were 106, 110, and 137 nm, respectively [149]. Therefore, the average size of the AgNPs decreased as the concentration of the *C. orbiculate* extract increased, which was attributed to the presence of more reducing agents and a faster AgNO_3_ reduction to form AgNPs. MIC and minimum bactericidal concentration (MBC) against MRSA were 40 μg/mL and 80 μg/mL, respectively. Lichen extracts were used as bioreducing agents to form AgNPs of 1–40 nm sizes in a study by Alqahtani et al. (2020) [150]. AgNPs inhibited the growth of both tested Gram-negative and Gram-positive multi-drug-resistant (MDR) strains with MIC and MBC values ranging between 0.019–0.156 and 0.039–0.625 mg/mL, respectively. Moorthy et al. (2021) produced AgNPs using aqueous and ethanolic bitter gourd (*M. charantia*) extracts (A-BG-AgNPs and E-BG-AgNPs, respectively) [152]. They found that E-BG-AgNPs were much smaller in size and showed greater agglomeration than the aqueous ones. A-BG-AgNPs showed better antibacterial performance than E-BG-AgNPs and both types of NPs produced morphological changes in *E. coli*, *S. aureus*, and *A. baumanii* cells.

Some studies used plant extracts for the biosynthesis of MNPs other than silver. Asghar et al. (2020) synthesized iron, copper, and silver NPs using *S. cumini* leaves extract [155]. The average diameters of Fe-, Cu-, and Ag-NPs were 58, 45, and 32 nm, respectively. Moreover, the antibacterial properties were found to be Ag- > Cu- > Fe-NPs, which showed that the size of NPs is an important factor in the effect of antibacterial activity, as stated in Table 2. Ssekatawa et al. (2022) used *C. sinensis* extract (CSE) and *P. africana* bark extract (PAE) to synthesize CuONPs [159]. The mean diameter for CSE and PAE NPs was 6 and 8 nm, respectively. Their antibacterial activity was significantly more extensive in MRSA, with lower MIC and MBC (30 μg/mL and 125 μg/mL, respectively) compared to MDR *E. coli* and *K. pneumoniae*. Palladium NPs with a diameter of 8.7 nm were synthesized using brown seaweed *P. boryana* extract in a study by Sonbol et al. (2021). They found that phytochemical compounds, such as tricosanoic acid, 2-methoxymethyl ester, 2-palmitoylglycerol, oleic acid chloride, oleic acid glycidyl ester, glycol stearate, monoolein, 9,12-octadecadienoyl chloride and oleic acid, 3-hydroxypropyl ester, were involved in the surface capping and stabilization of PdNPs. PdNPs were capable to inhibit Gram-positive and Gram-negative species and cause damage to the bacterial cell membrane permeability [160]. 

AgNPs stand out as the most used NPs, bringing together more than half of the total reports in Table 4. AgNPs have attracted a lot of attention in recent years, as evidenced by the significant demand for and investment in associated research [164]. AgNPs have seen steady market growth over the past 15 years, with an estimated 500 tons of NPs produced annually to meet demands across various industries. The research of their biological activity and safety, as well as the clarification of their precise mechanisms of action, have become matters of concern due to the rise of the NPs market globally [165].

The majority of the selected studies focus on DR strains of S. aureus, probably due to its use as a model bacteria in scientific research, as previously stated. It is followed by DR *K. pneumoniae* and DR *E. coli* strains, which are both common types of bacteria that can cause a range of infections, including urinary tract infections, pneumonia, and sepsis [48]. Some of the studies observed that Gram-negative bacteria showed greater sensitivity to NPs than Gram-positive ones [150,156,160,161], while other studies found the opposite case [148,159]. DR *P. aeruginosa* was used in various studies and its inhibition was especially remarkable compared to other ARB. The lower susceptibility of Gram-negative bacteria to the action of MNPs could be explained by cellular differences. Gram-positive bacteria have a thicker cell wall and more peptidoglycan, so they can become more resistant to the action of metal ions. Furthermore, the presence of negatively charged lipopolysaccharides in the cell wall of Gram-negative bacteria can promote the adhesion of MNPs [166].

Antibacterial activity was also shown to be dose-, size-, and shape-dependent [150]. Smaller MNPs are associated with an easier anchoring and penetration into bacterial cells. Furthermore, rod shape MNPs are related to a greater antibacterial capacity since they provide greater surface area [155]. The Z-potential is also a key parameter when determining antimicrobial activity. It has been observed that MNPs bind more efficiently to bacteria with a more negative Z-potential, which would also explain the greater susceptibility of Gram-negative bacteria [167]. 

In contrast to NFs, all studies of MNPs found used microdilution techniques to determine the MIC of the nanomaterials against ARB. These methods facilitate the comparison between different studies and their reproducibility.

## 6. Other Plant-Based Nanomaterials

Some studies used phytochemicals for synthesis or incorporation into nanomaterials other than NPs and NFs, which are summarized in Table 5. Qamar et al. (2020) synthesized CuO nanorods (NRs) via green synthesis using aqueous extracts of Momordica charantia. The synthesized NRs had a mean diameter of 61.48 nm in diameter and a length of 400–500 nm. In addition, CuO NRs were able to significantly inhibit MDR *B. cereus*, *Corynebacterium xerosis*, *E. coli*, *K. pneumoniae*, *Proteus vulgaris*, *P. aeruginosa*, *S. aureus*, *Staphylococcus epidermidis*, *Streptococcus mutans*, *S. pyogenes*, and *Streptococcus viridans* with the highest efficacy being observed against MDR *Bacillus cereus* [168]. ZnO–CuO nanocomposites were biosynthesized using *Calotropis gigantea* extract in a study by Govindasamy et al. (2021). The rod-shaped binary NPs had a diameter of 7.5 nm and a length of 8.1 nm. In addition, they were able to inhibit ARB *K. pneumoniae* and *P. aeruginosa* and MRSA with MIC values of 0.625, 0.156, and 0.156 mg/mL, respectively [169]. Azizi et al. (2017) fabricated hydrogel beads based on κ-Carrageenan loading biosynthesized Ag-NPs using *Citrullus colocynthis* seed extract [170]. The mean diameter of the synthesized NPs was 23 nm, while dried bio-nanocomposite hydrogel beads were spherical with a diameter of about 1 mm. The bio-nanocomposite hydrogel showed an inhibition zone of 11 mm against MRSA. 

## 7. Future Perspectives and Conclusions

The need to find new antibacterial agents that are effective against ARB is imperative. In this review, plant extracts and phytochemicals have been shown to play an important role in this matter in combination with nanotechnology. Polymeric NFs and NPs are valuable tools to encapsulate these compounds, ensuring their stability and controlled release, protecting them from degradation. On the other hand, the synthesis of MNPs with these compounds through the so-called “green synthesis” represents a more environmentally friendly approach that allows these tools to be used to fight ARB while limiting the use of toxic solvents and consumption of energy.

Many types of nanomaterials loaded or synthesized with a wide variety of natural compounds have shown activity against ARB. Among the polymers most used for the synthesis of NPs is CS, which shows intrinsic antibacterial properties and whose green synthesis has been recently described. In the case of NFs, the most used polymers are PCL and PVP, two biocompatible polymers. Silver NPs have been shown to be the most widely used MNPs for their study against ARB. It has been seen that the modulation of the properties of these nanocomposites (size, surface area, chemistry, porosity, etc.) can affect their antimicrobial activity.

Curcumin appears to be the most promising phytochemical for use in future developments, as it has been identified in three distinct studies in combination with electrospun NFs. The utilization of *A. vera* extract in two separate studies for the green synthesis of MNPs is also noteworthy. However, the effects of plant extracts are highly variable and dependent on several factors, such as their composition, which in turn can vary based on the extraction/purification method and the original raw materials used. These variables contribute to the multitude of antibacterial mechanisms that they possess, thereby rendering it challenging to identify any single extract as more significant than the others. Further research is needed in this direction to fully understand the mechanisms of action and potential limitations of this approach, evaluate the safety and toxicity of these combined phytochemical-nanomaterials, move on to the next phase of in vivo studies to discover the real therapeutic potential of these new biomedical tools, and be able to apply them onto practical biomedical applications. The fact that most of the studies found on this matter date from the last 5 years indicates the novelty and promising future of this technology, which can be decisive when developing tools to fight ARB. The innovativeness of this method explains the limited amount of information regarding the scale-up of green-synthesized nanomaterials. The next research steps should focus on optimizing various synthesis parameters to achieve the maximum yield possible while maintaining their desirable properties in the upscale process.

## Figures and Tables

**Table 1:** Electrospun polymer NFs loaded with phytochemicals against ARB.

Polymer	Phytochemical	Diameter (nm) *	Electrospinning	Antibacterial Activity *	Reference
Gelatin	*Phaeodactylum tricornutuen* extract	700	Blend	99.9% inhibition (MRSA)	[77]
PCL/gelatin	*Gymnema sylvestre* LE	302–340	Coaxial	ZOI 17.1 mm (MRSA)	[78]
PCL/gelatin	*Melia dubia* extract	256	Blend	ZOI 23 mm (MRSA)	[79]
PCL/PVP	Curcumin	880–740	Coaxial	37% inhibition (MRSA)	[80]
PVA	Myrrh extract	220	Blend	ZOI 13.33 mm (DR *S. aureus*)	[81]
	*Thymus vulgaris* extract	167	Blend	ZOI 10 mm (MRSA)	[82]
	*Salvia officinalis folium* extract	143		ZOI 10 mm (MRSA)	
	*Hyperici herba* extract	137		ZOI 10 mm (MRSA)	
PVA/CS	Curcumin	125	Blend/Coaxial	92% inhibition after 6 days (MRSA)	[83]
PVP	Emodin	692	Coaxial	Growing ZOI (MRSA)	[84]
P(HEMA)	Curcumin	20–110	Blend	ZOI 17 mm (MRSA), 18 mm (ESBL *Escherichia coli*)	[85]
Silk fibroin/PEO	Manuka honey	843–2229	Blend	ZOI 0.7–6.7 mm (MRSA)	[86]

DR: drug-resistant; ESBL: extended-spectrum beta-lactamases; LE: leaf extract; MRSA: methicillin-resistant *Staphylococcus aureus*. PCL: polycaprolactone; PEO: polyethilene oxide; PVA: polyvinyl alcohol; PVP: polyvinylpyrrolidone; P(HEMA): poly(2-hydroxyethyl methacrylate); ZOI: zone of inhibition. * Mean values or a range of values are indicated in studies employing various conditions or concentrations.

**Table 2:** Parameters affecting antimicrobial activity of polymeric NPs.

Parameters	Effects	References
Crosslinking	Crosslinked NPs may be more resistant to degradation and may release the antimicrobial agent more slowly.	[119]
Micellization	High critical micelle concentration can lead to higher antimicrobial activity due to the greater activity of the polymeric chains as free molecules in solution.	[120]
Molecular weight	High molecular weight polymers have shown greater antimicrobial activity against Gram-negative bacteria, due to the entrapment of the polymers by the peptidoglycan layer.	[121]
Polymer type and concentration	Some polymers, such as CS or PEI, have intrinsic antimicrobial activities and higher concentration may lead to a greater antimicrobial effect.	[122]
Size	Smaller sizes can enhance antimicrobial activity due to internalization to bacterial cells.	[123]
Surface area	Larger surface-to-volume NPs provide more active sites for bacterial interaction.	[124]
Surface chemistry	Type and density of functional groups in NPs surfaces can affect their antibacterial capacity by influencing their interactions with the bacterial cell surface.	[125]
Surface charges	Cationic charges increase antibacterial activity due to interaction with bacterial cell walls.	[126]

CS: chitosan; NPs: nanoparticles; PEI: polyethyleneimine.

**Table 3:** Polymeric NPs loaded/synthesized with phytochemicals against ARB.

Polymer	Phytochemical	Synthesis	Diameter (nm) *	Antibacterial Activity *	Reference
CS	Cardamom EO	Ionic gelation	50–100	Growth control for 2 days (MRSA, ESBL *E. coli*)	[127]
CS	*Eucalyptus globulus* LE	Green synthesis	7–10	ZOI of 12–30 (MDR *Acinetobacter baumannii*)	[128]
CS/HPMC	*Schinopsis brasiliensis* LE/Ceftriaxone	Polyelectrolytic complexation (coacervation)	150–500	MIC of 15 µg/mL (ESBL, KPC)	[129]
PLA/PVA	*Pistacia lentiscus* var. *chia* EO	Solvent evaporation	240–665	MIC higher than 3.4 mg/mL (DR *Bacillus subtilis* sub. *spizizenii*)	[130]

CS: chitosan; DR: drug-resistant; EO: essential oil; ESBL: extended-spectrum beta-lactamases; HPMC: hydroxypropyl methylcellulose; LE: leaf extract; MDR: multi-drug-resistant; MRSA: methicillin-resistant S. aureus; PLA: polylactic acid; PVA: polyvinyl alcohol; ZOI: zone of inhibition. * Mean values or a range of values are indicated in studies employing various conditions or concentrations.

**Table 4:** Green-synthesized MNPs using phytochemicals against ARB.

NPs	Phytochemical	Diameter (nm) *	MIC (µg/mL) *	Reference
AgNPs	*Aloe vera* extract	38.9	4.9–9.8 (KPC)	[147]
	*Cinnamomum tamala* LE	10–12	12.5 (MDR *E. coli*), 10 (MDR *K. pneumoniae*, 12.5 (MDR *S. aureus*)	[148]
	*Cotyledon orbiculate* LE	106–137	40 (MRSA)	[149]
	*Flavopunctelia flaventior* powder	69	0.156 (MRSA), 0.078 (VRE), 0.019 (MDR *Pseudomonas aeruginosa)*, 0.078 (MDR *E. coli*)	[150]
	*Mespilus germanica* LE	17.6	6.25–100 (MDR *K. pneumoniae*)	[151]
	*Momordica charantia* extract	9.6–16.4	4 (CR *A. baumannii)*, 4 (IR *A. baumannii*)	[152]
	*Periploca hydaspidis* extract	68.6–114.2	10 (MDR *K. pneumoniae),* 10–20 (MDR *S. aureus*), 10 (MDR *E. coli),* 5 (MRSA)	[153]
	*Stenocereus queretaroensis* PE	60–200	0.313 (MRSA)	[154]
	*Syzygium cumini* LE	10–15	8 (MRSA), 20 (VRSA)	[155]
	*Vaccinium macrocarpon* powder	1.4–8.6	18.3–39.5 (MRSA), 9.9–12.7 (MDR *P. aeruginosa*)	[156]
	*Xanthoria parietina* powder	145	0.078 (MRSA), 0.156 (VRE), 0.039 (MDR *P. aeruginosa)*, 0.156 (MDR *E. coli*)	[150]
AuNPs	*Anabaena spiroides* extract	80	25 (MDR *Klebsiella oxytoca*), 30 (MDR *Steptococcus pyogenes*), 20 (MRSA)	[157]
	*Punica granatum* extract	39.4	15.6 (MRSA)	[158]
CuNPs	*Syzygium cumini* LE	30–31	14 (MRSA), 16 (VRSA)	[155]
CuONPs	*Camellia sinensis* extract	61	125 (CREC), 125 (CRKP), 30 (MRSA)	[159]
	*Prunus africana* BE	68	125 (CREC), 125 (CRKP), 30 (MRSA)	
FeNPs	*Syzygium cumini* LE	40–46	11 (MRSA), 13 (VRSA)	[155]
PdNPs	*Padina boryana* extract	8.7	125 (MDR *S. aureus*), 62.5 (MDR *E. fergusonii*), 62.5 (MDR *A. pittii*), 62.5 (MDR *P. aeruginosa*), 62.5 (MDR *A. enteropelogenes*), 125 (MDR *P. mirabilis*)	[160]
TeNPs	*Aloe vera* extract	20–60	11.61 (MRSA), 3.53 (MDR *E. coli*)	[161]
ZnONPs	*Acacia nilotica* extract	94	0.45 (KPC)	[162]
	*Bougainvillea* FE	10–50	128 (MRSA), 128 (MREC)	[163]

BE: bark extract; CR: colistin-resistant; CREC: carbapenem-resistant *E. coli*; CRKP: carbapenem-resistant *K. pneumoniae*; FE: flower extract; IR: imipenem-resistant; KPC: *K. pneumoniae* carbapenemase; LE: leaf extract; MDR: multi-drug-resistant; MREC: methicillin-resistant *E. coli*; MRSA: methicillin-resistant *S. aureus*; NPs: nanoparticles; PE: peel extract; VRE: vancomycin-resistant *Enterococci*; VRSA: vancomycin-resistant *S. aureus*. * Mean values or a range of values are indicated in studies employing various conditions or concentrations.

**Table 5:** Nanomaterials other than NPs or NFs manufactured using phytochemicals with antimicrobial activity against ARB.

Nanomaterial	Phytochemical	Mean Size (nm)	Synthesis	Antibacterial Activity	Reference
CuO NRs	*Momordica charantia* FE	61.5 × 450	Green synthesis	ZOI of 28. 66 (MDR *S. aureus, S. mutans, C. xerosis)*, 25.66 (MDR *E. coli*, *P. aeruginosa*, * S. pyogenes)*, 27.33 (MDR *S. viridans*), 23 (MDR *S. epidermidis*), 31.66 (MDR *B. cereus*), 24.66 (MDR *K. pneumoniae*) and 26.33 (MDR *P. vulgaris*) mm	[168]
κ-Carrageenan/AgNPs hydrogel beads	*Citrullus colocynthis* SE	25	Green synthesis/Blending	ZOI of 11 mm (MRSA)	[170]
ZnO–CuO nanocomposites	*Calotropis* *gigantea* extract	8.1 × 7.5	Green synthesis	MIC of 0.16 (MDR *P. aeruginosa* and MRSA) and 0.63 (MDR *K. pneumoniae*)	[169]

FE: flower extract; MDR: multi-drug-resistant; MIC: minimum inhibitory concentration; MRSA: methicillin-resistant *S. aureus*; NRs: nanorods; SE: seed extract; ZOI: zone of inhibition.

## Data Availability

Not applicable.
